# The Prognosis of Cardiac Origin and Noncardiac Origin in-Hospital Cardiac Arrest Occurring during Night Shifts

**DOI:** 10.1155/2016/4626027

**Published:** 2016-09-27

**Authors:** Yuan-Jhen Syue, Jyun-Bin Huang, Fu-Jen Cheng, Chia-Te Kung, Chao-Jui Li

**Affiliations:** ^1^Department of Anesthesiology, Kaohsiung Chang Gung Memorial Hospital, Chang Gung University College of Medicine, Kaohsiung, Taiwan; ^2^Department of Emergency Medicine, Kaohsiung Chang Gung Memorial Hospital, Chang Gung University College of Medicine, Kaohsiung, Taiwan

## Abstract

*Background.* The survival rates of in-hospital cardiac arrests (IHCAs) are reportedly low at night, but the difference between the survival rates of cardiac origin and noncardiac origin IHCAs occurring at night remains unclear.* Methods.* Outcomes of IHCAs during different shifts (night, day, and evening) were compared and stratified according to the etiology (cardiac and noncardiac origin).* Result.* The rate of return of spontaneous circulation (ROSC) was 24.7% lower for cardiac origin IHCA and 19.4% lower for noncardiac origin IHCA in the night shift than in the other shifts. The survival rate was 8.4% lower for cardiac origin IHCA occurring during the night shift, but there was no difference for noncardiac origin IHCA. After adjusting the potential confounders, chances of ROSC (aOR: 0.3, CI: 0.15–0.63) and survival to discharge (aOR: 0.1; CI: 0.01–0.90) related to cardiac origin IHCA were lower during night shifts. Regarding noncardiac origin IHCA, chances of ROSC (aOR: 0.5, CI: 0.30–0.78) were lower in the night shift, but chances of survival to discharge (aOR: 1.3, CI: 0.43–3.69) were similar in these two groups.* Conclusion.* IHCA occurring at night increases mortality, and this is more apparent for cardiac origin IHCAs than for noncardiac origin IHCA.

## 1. Introduction

According to the Get with the Guidelines- (GWTG-) Resuscitation registry, the outcomes following in-hospital cardiac arrest (IHCA) have recently improved [1, 2]. This has been attributed to the improvement in the management of acute coronary syndrome (ACS), which also results in a decline in the numbers of IHCAs of cardiac origin [1–4]. However, several studies show that the survival rates of IHCAs remain low during nights [5–10]. The reason for poor prognosis of IHCA during the night shift might be multifactorial, potentially including healthcare staff and hospital staff as well as the differences in the etiologies of cardiac arrest. Matot et al. reported that unwitnessed cardiac arrest is more prevalent during a night shift. They reported that resuscitation during this shift may not be associated with poorer outcomes, independent of the witnessed status [5]. To our knowledge, studies on the association between the etiology of cardiac arrest in different shifts and patient prognosis are relatively scarce. In this study, to understand the distribution of the etiologies of IHCA in different shifts and their influence on clinical outcome, we compared the prognoses in different shifts by stratifying the etiologies of IHCA into cardiac origin and noncardiac origin. We aimed to determine whether the etiologies of IHCA influence the patient's prognosis during a night shift.

## 2. Materials and Methods

### 2.1. Study Design

This retrospective study was approved by the Chang Gung Medical Foundation Institutional Review Board. Based on the understanding that all data in the patient and physician records used in the analyses have been anonymized and deidentified, the ethics committee approved the research protocol with a waiver of informed consent.

### 2.2. Study Setting

This study was conducted across the Kaohsiung Chang Gung Memorial Hospital, a tertiary teaching hospital, with 2715 beds, including 1388 beds in the adult general ward and 203 beds in the adult intensive care unit (ICU). The resuscitation team in the study included the physicians and nurses on duty. An emergency response team at the institute provided round-the-clock aid for those in need. All members of the resuscitation team were certified in advanced cardiac life support (ACLS). The primary care nurse was responsible for recording all of the resuscitation efforts, procedures, and medications on a standardized resuscitation data form during the event.

### 2.3. Participants

Patients aged 18 years or older who experienced a cardiac arrest requiring cardiopulmonary resuscitation (CPR) or defibrillation in general wards were included in the analysis. Only index events were included. An index event is defined as the first arrest for patients having more than 1 arrest during the same hospitalization period. Exclusion criteria included (1) patients receiving only resuscitation drugs or positive pressure ventilation without chest compression or defibrillation, (2) patients receiving palliative treatment or signing “do not resuscitate (DNR)” order, and (3) patients with preexisting tracheostomy or chronic mechanical ventilation.

### 2.4. Study Protocol

Patient data were drawn from in-hospital cardiac arrest registry of the Kaohsiung Chang Gung Memorial Hospital from January 2008 to December 2011. Event details were recorded by the responding nurses and primary physician of the resuscitation team who had attended the event. The data contains variables derived from Utstein data reporting guidelines for in-hospital cardiac arrest [11–13]. The patient outcome after cardiac arrest occurring during the night shift (00:00–8:00) was compared with that during the day (8:00–16:00) or evening (16:00–24:00) shift.

### 2.5. Measures

Independent variables comprised time of event, basic patient characteristics, comorbidities of Charlson Comorbidity Index (CCI) [14], main morbidity on admission, admission department, interventions before IHCA, first documented cardiac rhythm, discovery status at the time of event, and possible cause of cardiac arrest. The discovery status at time of event was included if there was a deteriorating disease course, if events were witnessed, and if there was bystander CPR. A deteriorating disease course was defined as a respiratory rate of ≤5 or ≥32 breaths/min, a pulse rate of ≤40 or ≥140 beats/min, a systolic blood pressure of <90 mmHg, and a sudden fall in the level of consciousness by 2 or more Glasgow Coma Scale (GCS) points within 8 hours before cardiac arrest [15]. The possible cause of cardiac arrest was categorized into cardiac origin and noncardiac origin. To determine the etiology of the cardiac arrest, 2 experienced EPs independently selected one of the following possible causes: cardiac, respiratory, cerebral, or metabolic causes, sepsis, exsanguination, hypothermia, drug overdose, or others [16]. Reviewers also consulted the initial treating physicians, if available, to clarify details and possible etiologies. If opinions were inconsistent, the 3rd experienced EP would be consulted, and a final decision was made after group discussion. The outcome is the return of spontaneous circulation (ROSC) for more than 20 minutes with no further need for chest compressions and survival to hospital discharge. Neurological outcome was determined using Cerebral Performance Category (CPC) score which allocates a score of 1 for good cerebral performance, 2 for moderate performance, 3 for poor performance, 4 for comatose or vegetative status, and 5 for brain death. In this study, patients with a score of 1 or 2 were defined as those with favorable neurological outcomes.

### 2.6. Data Analysis

For continuous variables, the data were summarized as the mean and standard deviation (SD) and analyzed by Student's* t*-test. The distributions of categorical variables were summarized as numbers and percentages, and Chi-square test was used to evaluate the associations between outcome groups. In the multivariate analyses, binary logistic regression models were applied to assess the effect of the night shift on documented patient outcomes to adjust for the potential confounding factors including patient's age, sex, CCI, first documented cardiac rhythm, witnessed cardiac arrest, and bystander CPR. Effects were estimated in terms of adjusted odds ratios (aORs) and the corresponding 95% confidence intervals (CIs). Results were considered statistically significant for two-tailed *p* < 0.05. The statistical analysis was conducted using SPSS version 12.0 (SPSS, Chicago, IL) for Windows.

## 3. Result

### 3.1. Patient Demographics

During the study period, 544 adult patients with IHCA occurring in general wards were analyzed. A total of 331 (60.8%) patients developed cardiac arrest during the day or evening shift, and 213 (39.2%) patients developed cardiac arrest during the night shift.  Table 1 reveals the patients' age, sex, predisposing diseases, and primary cause of admission in the two study groups, which shows no significant difference between study groups.

### 3.2. Event Characteristics

Table 2 reveals the characteristics of cardiac arrest. There was no significant difference between the two study groups in the distribution of the wards in which the arrest occurred and interventions prior to cardiac arrest. The percentages of patients who developed cardiac arrest with the predictable deteriorated disease cause were similar in the two study groups. The distributions of cardiac origin and noncardiac origin of cardiac arrest were also similar in the two study groups. However, the first documented cardiac rhythm was different in the two study groups. Patients who developed cardiac arrest during the night shift were more likely to be found with asystole rhythm. In contrast, fewer patients were witnessed to have collapsed and received bystander CPR during the night shift.

**Figure 1:**
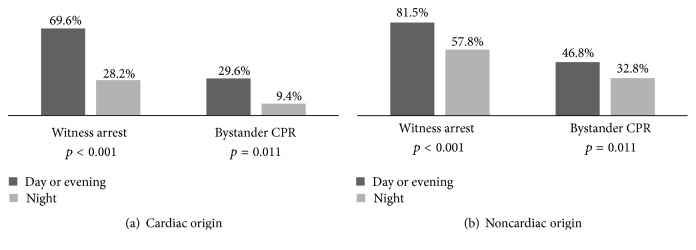
Percentage of witnessed event and bystander CPR at the time of the event per shift: (a) cardiac origin in-hospital cardiac arrest and (b) noncardiac origin in-hospital cardiac arrest.

**Figure 2:**
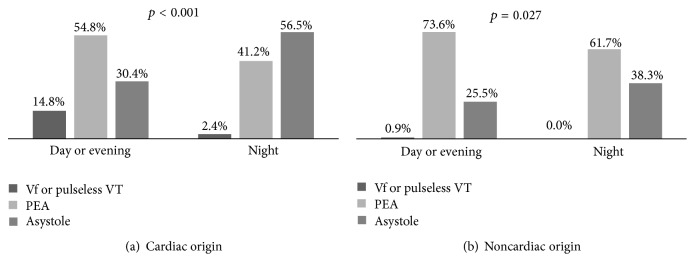
Percentage of initial rhythm at the time of the event per shift: (a) cardiac origin in-hospital cardiac arrest and (b) noncardiac origin in-hospital cardiac arrest. VF: ventricular fibrillation; VT: ventricular tachycardia; PEA: pulseless electrical activity.

After stratifying the causes of cardiac arrest, in cardiac origin IHCA, the incidence of witness arrest and bystander CPR in the night shift was less by 41.4% and 20.2%, respectively, than in morning and evening shifts combined (Figure 1(a)). In noncardiac origin IHCA, the chance was 23.7% and 14.0% less, respectively (Figure 1(b)). The cardiac origin IHCA during night shifts presented 26.1% more asystole rhythm and 12.4% lesser shockable rhythm (VF and pulseless VT) in the initially documented cardiac rhythm compared to those during morning and evening shifts combined (Figure 2(a)). For noncardiac origin IHCA, they were 12.8% more and 0.9% less, respectively (Figure 2(b)).

### 3.3. Outcome of Cardiac Arrest

**Figure 3:**
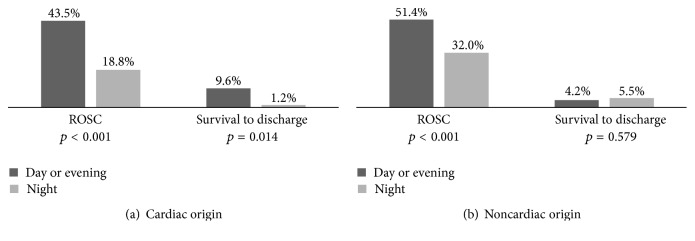
Percentage of ROSC and survival to discharge of the event per shift: (a) cardiac origin in-hospital cardiac arrest and (b) noncardiac origin in-hospital cardiac arrest.

The overall ROSC rate and survival to discharge rate for the whole study group were 40.1% and 5.1%.  Figure 3 reveals the outcomes of cardiac arrest stratified by cause of cardiac arrest during each of the shifts. The ROSC rate of cardiac origin IHCA was 24.7% lower during the night shift than during the day or evening shift (*p* < 0.001), while it was 19.4% lower for noncardiac origin IHCA (*p* < 0.001). The survival rate of cardiac origin IHCA was 8.4% lower during the night shift than during the day or evening shift (*p* = 0.014); however, the survival rate of noncardiac origin IHCA showed no difference compared to that in the other shifts (*p* = 0.579).

Controlling for the potential confounders with multivariate logistic regression including patients' age, sex, CCI, first documented cardiac rhythm, witnessed event, and bystander CPR, the adjusted odds ratio of ROSC and survival to discharge between the night shift and day or evening shift combined in cardiac origin IHCA and noncardiac origin IHCA are demonstrated in Table 3. The chance of ROSC (aOR: 0.3, CI: 0.15–0.63) and survival to discharge (aOR: 0.1; CI: 0.01–0.90) of cardiac origin IHCA was lower during the night shift than during the day or evening shift. For noncardiac origin IHCA, the chance of ROSC (aOR: 0.5, CI: 0.30–0.78) was lower during the night shift than during the day or evening shift, but the chances of survival to discharge (aOR: 1.3, CI: 0.43–3.69) were similar in the two study groups.

**Figure 4:**
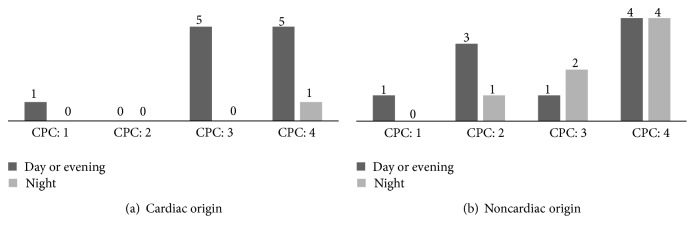
The distribution of patients with survival to discharge in the Cerebral Performance Category score: 1 for good cerebral performance, 2 for moderate performance, 3 for poor performance, and 4 for comatose or vegetative status.

Figure 4 displays the distribution of patients' survival to discharge in the Cerebral Performance Category score. Among patients with either cardiac or noncardiac origin IHCA, only 1 patient in each group had good cerebral performance during the day or evening shift and the others had poor performance, were comatose, or had vegetative status. Three patients during the day or evening shift and one patient during the night shift had moderate performance. The others had poor performance, were comatose, or had a vegetative status.

## 4. Discussion

Previous studies reported that survival rates from in-hospital cardiac arrest are lower during nights [5, 6]. In this study, we attempted to compare the prognoses of patients developing IHCA during night, morning, and evening shifts with cardiac origin cause and noncardiac origin cause. We found that, for cardiac origin IHCA, both ROSC and survival to discharge were significantly lower for patients who were resuscitated during the night shift when compared to morning and evening shifts combined. In noncardiac origin IHCA, ROSC was significantly lower for patients developing cardiac arrest in the night shift, but chances of survival to discharge were similar in the two study groups. The demographic characteristics, departments of events, interventions before events, and the distribution of cardiac origin and noncardiac origin IHCA of the study population undergoing resuscitation in the various shifts were similar. However, there were more unwitnessed arrests, higher incidence of asystole, and less shockable rhythm (VF and pulseless VT) during the night shifts. After adjusting these potential confounding factors with multivariate logistic regression, resuscitation during night shifts had lower chances to achieve ROSC in both cardiac origin IHCA and noncardiac origin IHCA, but night shifts had lower chances of survival to discharge in cardiac origin IHCA, which is not evident in noncardiac origin IHCA. It is believed that the most likely reason for the incidence of ROSC and survival to discharge being lower at night is the fact that more arrests were unwitnessed, and it could be proven by the fact that asystole was more frequent at night. However, after adjusting for the cardiac rhythm, the outcome of IHCA at night was still poorer. It might be explained by the delay of activation of resuscitation team and limitation of hospital facilitation such as percutaneous transluminal coronary angioplasty or extracorporeal membrane oxygenation.

According to previous studies, one of the predictors of good prognosis after IHCA is the event being witnessed [3, 4, 17–20]. In this study, the witnessed events were lower during night shifts than during morning and evening shifts combined by 31.3%. The lower rate was more obvious in cardiac origin IHCA (41.4%) than in noncardiac origin IHCA (23.7%). Bystander CPR rate was also lower in the night shifts by 17.3%. Again, the lower rate was more remarkable in cardiac origin IHCA (20.2%) than in noncardiac origin IHCA (14.0%). These might explain why the lower rate of ROSC and survival to discharge were more marked in cardiac origin IHCA (24.7% and 8.4%, resp.) than in noncardiac origin IHCA (19.4% and no difference, resp.). The underlying cause for lower rate of witnessed events and bystander CPR for cardiac origin IHCA compared to noncardiac origin IHCA may be due to patients making less noise during cardiac origin IHCA as compared to those with noncardiac origin IHCA. Patients that develop noncardiac origin IHCA usually show signs of choking or major bleeding such as gastrointestinal bleeding or upper aerodigestive tract tumor bleeding, noises from which help alert the patient's caregiver to identify the events. Another evidence for delayed discovery of cardiac origin IHCA was the initial documented cardiac rhythm. The increased rate of asystole rhythm for IHCA at night was higher for cardiac origin IHCA (26.1%) than for noncardiac origin IHCA (12.8%), which may contribute the poorer prognosis.

The overall rate of survival to discharge and favorable neurological outcome (with CPC score of 1 or 2) were conspicuously lower in this study than in others. According to the previous study, the survival to discharge rates increased from 13.7% in 2000 to 22.3% in 2009 and significant neurologic disability among survivors decreased from 32.9% to 28.1% in 2000 and 2009, respectively [1, 2]. One possible explanation was that the previous study included IHCA patients both in general wards and in ICUs. According to previous study, patients being monitored played an important role in good prognosis after IHCA [3, 4, 17–20]. Patient monitoring is more difficult in general wards than in ICUs. Besides, there are also differences in the healthcare economics, patient expectation, and provider practice patterns in Taiwan. Although do not resuscitate orders have started to be accepted by people in Taiwan, there were still some patients receiving CPR, with predictable deteriorating disease course in terminal diseases. The lower possibilities of successful resuscitation for these patients might also result in the poor prognosis.

## 5. Conclusion

This study offers further insight into the complex relationship between in-hospital resuscitation during the night shift and increased mortality, and this phenomenon is more obvious in cardiac origin IHCAs than in noncardiac origin ones.

## 6. Limitations

There were several limitations to this study. First, it was a single center study. Second, the retrospective nature of the study made it difficult to assemble data and judge the precise etiology of cardiac arrest by chart review because of the lack of objective information. Third, practice patterns in Taiwan differ in some ways that are quite remarkable compared to the US and other Western countries, particularly related to decisions regarding “do not resuscitate” order. We believe that these differences may influence the interpretation of the result by other medical systems. Fourth, this study could not trace the patients' outcomes after discharge, so no long-term mortality rate or quality of life was documented.

## Figures and Tables

**Table 1:** Patient demographics.

	Day/evening (331)		Night (213)		*p* value
*Age*	67.1	±15.17	68.1	±14.84	0.428
*Male*	196	59.2%	124	58.2%	0.817
*Charlson Comorbidity Score*	4.5	±2.57	4.8	±2.53	0.201
*Predisposing disease*					
Coronary artery disease	49	14.8%	30	14.1%	0.816
Congestive heart failure	53	16.0%	29	13.6%	0.446
Cerebral vascular disease	76	23.0%	52	24.4%	0.697
Chronic pulmonary disease	72	21.8%	48	22.5%	0.830
Chronic liver disease	72	21.8%	62	29.1%	0.052
Chronic kidney disease	127	38.4%	76	35.7%	0.527
DM	129	39.0%	79	37.1%	0.659
Malignancy	104	31.4%	67	31.5%	0.993
Hematologic disease	20	6.0%	12	5.6%	0.843
Psychiatric disorder	10	3.0%	3	1.4%	0.229
*Primary cause of admission*					
Cardiovascular disease	32	9.7%	14	6.6%	0.205
Cerebral vascular disease	13	3.9%	13	6.1%	0.246
Infection	106	32.0%	81	38.0%	0.150
Complication of liver cirrhosis	21	6.3%	23	10.8%	0.063
Complication of renal failure	22	6.6%	12	5.6%	0.634
Complication of DM	10	3.0%	2	0.9%	0.107
Malignancy	67	20.2%	36	16.9%	0.332
Hematologic disease	15	4.5%	4	1.9%	0.100

**Table 2:** Event characteristics.

	Day/evening (331)		Night (213)		*p* value
*Department of events*					
Internal medicine	297	89.7%	193	90.6%	0.469
Surgical medicine	24	7.3%	17	8.0%	
Other	10	3.0%	3	1.4%	
*Intervention before events*					
Oxygen therapy	163	49.2%	115	54.0%	0.280
Inhalation therapy	29	8.8%	28	13.1%	0.103
Vascular access	306	92.4%	201	94.4%	0.386
Antibiotics therapy	199	60.1%	128	60.1%	0.995
Inotropic agent	7	2.1%	2	0.9%	0.294
*First documented cardiac rhythm*					
Vf or pulseless VT	19	5.70%	2	0.90%	<0.001
PEA	222	67.10%	114	53.50%	
Asystole	90	27.20%	97	45.50%	
*Discovery status at time of event*					
Deteriorated disease course	189	57.1%	108	50.7%	0.144
Witnessed	256	77.3%	98	46.0%	<0.001
Bystander CPR	135	40.8%	50	23.5%	<0.001
*Possible cause of cardiac arrest*					
Cardiac origin	115	34.7%	85	39.9%	0.223
Noncardiac origin	216	65.3%	128	60.1%	

**Table 3:** Cardiac arrest outcomes by day/evening versus night.

Outcome	Day or evening shift	Night shift	
	Reference	aOR	95% CI
*Cardiac origin*			
ROSC	[1]	0.3^*∗*^	0.15~0.63
Survival to discharge	[1]	0.1^*∗*^	0.01~0.90
*Noncardiac origin*			
ROSC	[1]	0.5^*∗*^	0.30~0.78
Survival to discharge	[1]	1.3	0.43~3.69

^*∗*^Significant factor.

ROSC: return of spontaneous circulation.

aOR: adjusted odds ratio, for patient's age, sex, CCI, first documented cardiac rhythm, witnessed cardiac arrest, and bystander CPR.

## References

[1] Girotra S., Cram P., Spertus J. A. (2014). Hospital variation in survival trends for in-hospital cardiac arrest. *Journal of the American Heart Association*.

[2] Girotra S., Nallamothu B. K., Spertus J. A., Li Y., Krumholz H. M., Chan P. S. (2012). Trends in survival after in-hospital cardiac arrest. *The New England Journal of Medicine*.

[3] Larkin G. L., Copes W. S., Nathanson B. H., Kaye W. (2010). Pre-resuscitation factors associated with mortality in 49,130 cases of in-hospital cardiac arrest: a report from the National Registry for Cardiopulmonary Resuscitation. *Resuscitation*.

[4] Meaney P. A., Nadkarni V. M., Kern K. B., Indik J. H., Halperin H. R., Berg R. A. (2010). Rhythms and outcomes of adult in-hospital cardiac arrest. *Critical Care Medicine*.

[5] Matot I., Shleifer A., Hersch M. (2006). In-hospital cardiac arrest: is outcome related to the time of arrest?. *Resuscitation*.

[6] Peberdy M. A., Ornato J. P., Larkin G. L. (2008). Survival from in-hospital cardiac arrest during nights and weekends. *The Journal of the American Medical Association*.

[7] Brindley P. G., Markland D. M., Mayers I., Kutsogiannis D. J. (2002). Predictors of survival following in-hospital adult cardiopulmonary resuscitation. *Canadian Medical Association Journal*.

[8] Dumot J. A., Burval D. J., Sprung J. (2001). Outcome of adult cardiopulmonary resuscitations at a tertiary referral center including results of ‘limited’ resuscitations. *Archives of Internal Medicine*.

[9] Herlitz J., Bång A., Alsén B., Aune S. (2002). Characteristics and outcome among patients suffering from in hospital cardiac arrest in relation to whether the arrest took place during office hours. *Resuscitation*.

[10] Takeda Y., Mifune J., Taga K. (1989). Survival after sudden cardiac arrest in hospital. *Japanese Heart Journal*.

[11] Jacobs I., Nadkarni V., Bahr J. (2004). Cardiac arrest and cardiopulmonary resuscitation outcome reports: update and simplification of the Utstein templates for resuscitation registries: a statement for healthcare professionals from a task force of the International Liaison Committee on Resuscitation (American Heart Association, European Resuscitation Council, Australian Resuscitation Council, New Zealand Resuscitation Council, Heart and Stroke Foundation of Canada, InterAmerican Heart Foundation, Resuscitation Councils of Southern Africa). *Circulation*.

[12] Nadkarni V. M., Larkin G. L., Peberdy M. A. (2006). First documented rhythm and clinical outcome from in-hospital cardiac arrest among children and adults. *The Journal of the American Medical Association*.

[13] Peberdy M. A., Kaye W., Ornato J. P. (2003). Cardiopulmonary resuscitation of adults in the hospital: a report of 14720 cardiac arrests from the National Registry of Cardiopulmonary Resuscitation. *Resuscitation*.

[14] Charlson M. E., Pompei P., Ales K. L., MacKenzie C. R. (1987). A new method of classifying prognostic comorbidity in longitudinal studies: development and validation. *Journal of Chronic Diseases*.

[15] Hillman K. M., Bristow P. J., Chey T. (2001). Antecedents to hospital deaths. *Internal Medicine Journal*.

[16] Kürkciyan I., Meron G., Behringer W. (1998). Accuracy and impact of presumed cause in patients with cardiac arrest. *Circulation*.

[17] Brady W. J., Gurka K. K., Mehring B., Peberdy M. A., O'Connor R. E. (2011). In-hospital cardiac arrest: impact of monitoring and witnessed event on patient survival and neurologic status at hospital discharge. *Resuscitation*.

[18] Chan P. S., Spertus J. A., Krumholz H. M. (2012). A validated prediction tool for initial survivors of in-hospital cardiac arrest. *Archives of Internal Medicine*.

[19] Ebell M. H., Afonso A. M. (2011). Pre-arrest predictors of failure to survive after in-hospital cardiopulmonary resuscitation: a meta-analysis. *Family Practice*.

[20] Herlitz J., Bång A., Aune S., Ekström L., Lundström G., Holmberg S. (2001). Characteristics and outcome among patients suffering in-hospital cardiac arrest in monitored and non-monitored areas. *Resuscitation*.

